# Adaptive Benefits of Storage Strategy and Dual AMPK/TOR Signaling in Metabolic Stress Response

**DOI:** 10.1371/journal.pone.0160247

**Published:** 2016-08-09

**Authors:** Benjamin Pfeuty, Quentin Thommen

**Affiliations:** Laboratoire de Physique des Lasers, Atomes et Molécules, Université de Lille Sciences et Technologies, CNRS, Villeneuve d’Ascq, France; University of Erlangen-Nuremberg, GERMANY

## Abstract

Cellular metabolism must ensure that supply of nutrient meets the biosynthetic and bioenergetic needs. Cells have therefore developed sophisticated signaling and regulatory pathways in order to cope with dynamic fluctuations of both resource and demand and to regulate accordingly diverse anabolic and catabolic processes. Intriguingly, these pathways are organized around a relatively small number of regulatory hubs, such as the highly conserved AMPK and TOR kinase families in eukaryotic cells. Here, the global metabolic adaptations upon dynamic environment are investigated using a prototypical model of regulated metabolism. In this model, the optimal enzyme profiles as well as the underlying regulatory architecture are identified by combining perturbation and evolutionary methods. The results reveal the existence of distinct classes of adaptive strategies, which differ in the management of storage reserve depending on the intensity of the stress and in the regulation of ATP-producing reaction depending on the nature of the stress. The regulatory architecture that optimally implements these adaptive features is characterized by a crosstalk between two specialized signaling pathways, which bears close similarities with the sensing and regulatory properties of AMPK and TOR pathways.

## Introduction

To cope with environmental changes that impact their metabolism, living cells have evolved adaptive strategies consisting in sensing their extracellular or intracellular environment and regulating accordingly the activity of enzymes catalyzing metabolic reaction pathways. These strategic tasks involve only a few signaling pathways in spite of the huge number of enzyme-catalyzed metabolic pathways. In eukaryotes, the highly conserved AMPK (AMP-activated kinase) and TOR (target of rapamycine) families of protein kinase have crucial and numerous roles in nutrient and energy sensing, and in governing metabolic adaptations by regulating the expression and post-translational modifications of many metabolic enzymes [1–3]. Mammalian AMPK and its yeast and plant homologs Snf1 and SnRK1 are prone to be activated, allosterically or through phosphorylation, upon intracellular increases of AMP or ADP levels [4–6]. In turn, AMPK/Snf1/SnRK1 kinases tend to switch off anabolic pathways, including the biosynthesis of proteins, ribosomal RNA, carbohydrates or lipids while promoting their degradation through autophagy and fatty acid oxidation [7]. For its part, the TOR pathway is rather sensitive to intracellular levels of metabolites, especially amino acids, and promotes growth by activating regulating biosynthetic pathways at the level of both transcriptional and translational machinery [8–10]. Besides their opposite roles in regulating biosynthetic pathways, both signaling pathways nevertheless share the same inclination to activate certain processes such as glycolysis or mitochondrial oxidative metabolism. For the latter, TOR promotes PGC-1*α* [11], 4EBP dependent translational regulation [12] or TCA enzymes such as Glu dehydrogenase [13], and AMPK mediates as well the activation of mitochondrial enzymes mainly through pathways converging to PCG1*α*/p [14, 15].

The crosstalk between AMPK and TOR signaling in sensing various intracellular cues and in regulating diverse anabolic and catabolic pathways raises a number of theoretical issues. The issue of intracellular sensing raises a difficult problem as these sensors are embedded into a global feedback architecture [16, 17]. As well, the issue of regulatory logic has been mainly studied for unbranched metabolic pathways [18–21] but much less for coupled metabolic pathways that both cooperate and compete for the utilization of internal resources. Besides the detailed schemes of sensing and regulatory mechanisms, several general questions arise about the adaptive logic of cell metabolism: How do signaling and regulatory strategies depend on the nature, frequency, duration, amplitude or randomness of environmental perturbations? What are the minimal requirements and the precise mechanisms that confer an adaptive benefit upon storage metabolism? The present study aims to address most of these issues through a minimal modeling approach.

Diverse computational modeling approaches have been developed to study the regulation of cell metabolism [22]. These approaches are generally based on a dual-level description made of a metabolic reaction network and an enzyme regulatory network. First, constraint-based stochiometric models of genome-scale metabolic reaction network use steady-state assumptions and do not provide information on the enzymatic concentrations. Nevertheless, several extensions have attempted to overcome these limitations by incorporating a description of gene regulation [23], by considering enzyme costs and capacity constraints [24], by performing a timescale separation hypothesis [24], or by using sensitivity analysis [25]. Second, metabolic control analysis is a powerful framework to study the response properties of complex metabolic systems to small changes of the kinetic parameters, which can be used to derive the optimal linear feedback regulation to static perturbation of steady state [26], and can be extended to the cases of non-steady state trajectories [27] or of time-dependent changes of kinetic parameters [28]. Although these two main modeling frameworks are well-adapted to determine optimal flux balance in detailed metabolic reaction networks, they remain dependent on steady-state or quasi-steady-state assumptions or on small perturbation approximations. A third approach consists in using simplified models depicting generic motifs (unbranched or cyclic pathways) or a prototypical metabolism, which allows studying regulation in simple resource allocation problem such as the switch from one to another substrate [29, 30], the switch between respiratory and fermentation metabolism [31], or the evolution of regulatory complexity [32]. The total number of kinetic parameters in these models is usually low enough to allow for extensive parameter space exploration or for parameter optimization through evolutionary computation techniques, without necessarily requiring additional assumptions of steady state or of small enough environmental fluctuations.

In this paper, minimal modeling and evolutionary computation are exploited to investigate the regulated coordination of catabolic and anabolic processes, and to decipher the logic underlying the universal sensing and regulatory features of TOR and AMPK signaling pathways. For this purpose, we introduce a coarse-grained model of cell metabolism that recapitulates the main catabolic and anabolic pathways. Steady-state and perturbation analysis are first performed to identify the regulatory logic in response to very slow or very small perturbations. Evolutionary computation is then applied to investigate adaptive strategy to a large range of perturbation amplitude and frequency, and to obtain the optimal enzyme time course and regulatory parameters. The results and the closing discussion emphasize the coordinated roles of storage metabolism, internal sensing and regulatory crosstalk, for metabolic adaptation to dynamic and complex environments.

## Methods

**Fig 1:**
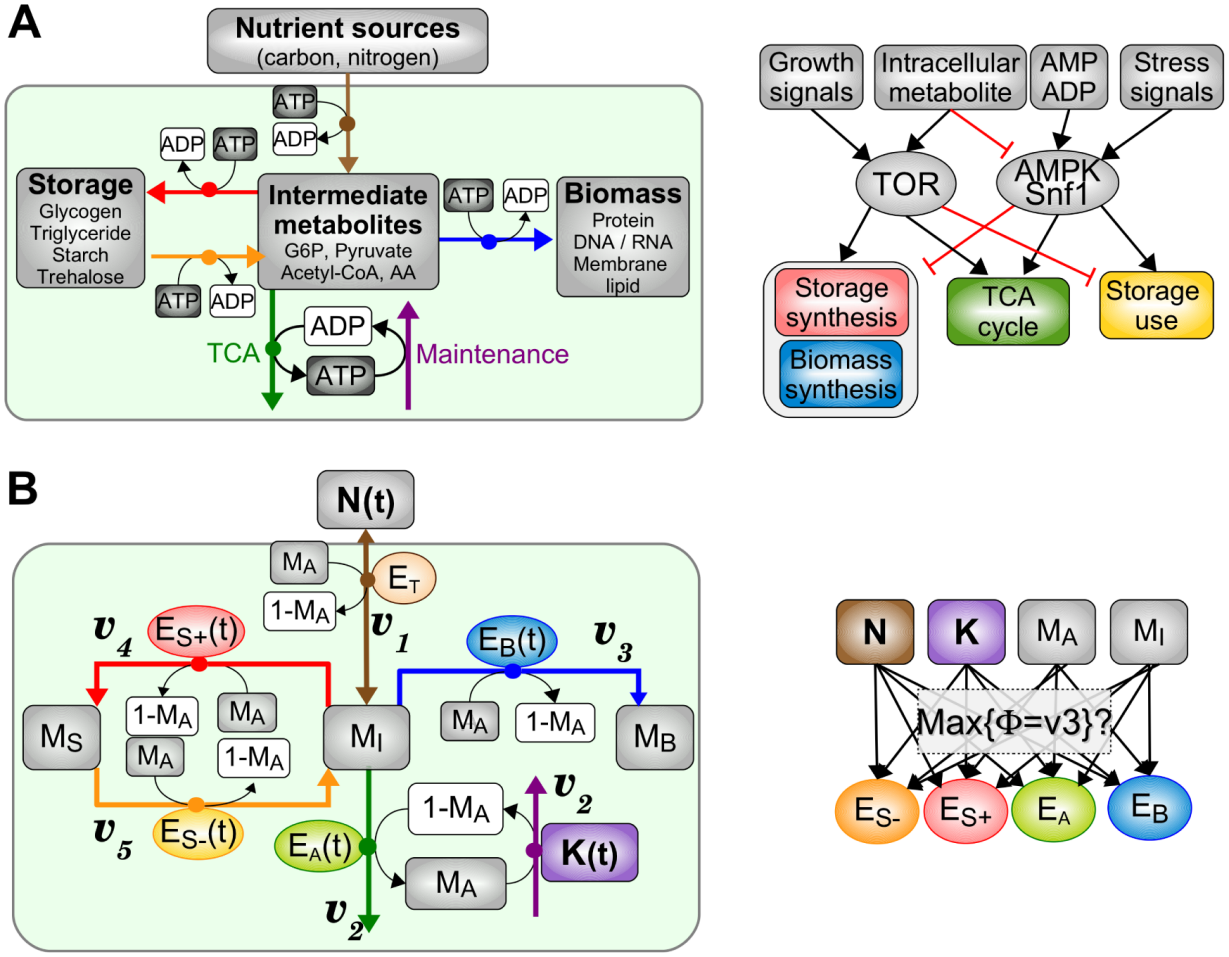
Coarse-grained description of cell metabolism. (A) Main anabolic and catabolic metabolic pathways regulated by TOR and AMPK/Snf1 signaling pathways in eukaryotic cells. (B) Corresponding model including metabolite concentration variables *M*_*i*_, enzyme-dependent rate coefficient variables *E*_*i*_ and varying resource *N* and demand *K*, organized into a metabolic network (left) and a signaling/regulatory network (right). The model objective is to identify the dynamic enzyme pattern *E*_*i*_(*t*) first, and then the signaling and regulatory architecture, such as the biomass production rate *v*_3_ called growth rate fitness Φ is maximized.

We consider a coarse-grained description of cell metabolism where nutrients are imported and catabolized into intermediate metabolites that can be either oxidized through the TCA cycle to produce ATP or utilized as precursors to build storage or biomass materials. In turn, ATP fuels most of the import, maintenance and biosynthetic processes (Fig 1A). Each of these coarse-grained processes are based on a chain of reactions that is regulated by a pool of enzymes and is characterized by a global energy budget in terms of ATP cost or gain. Such schematic model of regulated metabolism can be translated into a biochemical reaction rate model (Fig 1B and Table 1) where each macroprocess is modeled by a single reaction catalyzed by a single enzyme and consumes a given amount of ATP. The model comprises different classes of variables and parameters: (i) metabolic variables *M*_*j*_ where *j* = *I* for intermediate, *j* = *S* for storage, *j* = *B* for biomass and *j* = *A* for ATP; (ii) rate coefficient variables *E*_*j*_ for the *j*th reaction (*j* = *A*, *B*, *S*_+_, *S*_−_, *T*, *M*), which are typically proportional to enzyme concentrations assuming the linear regime of Michaelis-Menten kinetics; (iii) resource and demand variables, *N* and *K*; (iv) budget parameters *k*_*j*_ for the cost or gain in ATP of the *j*th reaction.

**Table 1:** Biochemical reactions and parameter values.

Nutrient transport	$N+k_{T}M_{A}+E_{T}\overset{*}{↔}M_{I}+k_{T}\left(1-M_{A}\right)+E_{T}$ $v_{1}=E_{T}M_{A}H\left(N-M_{I}\right)$
ATP production	*M*_*I*_ + *k*_*A*_(1 − *M*_*A*_) + *E*_*A*_ → *k*_*A*_*M*_*A*_ + *E*_*A*_ *v*_2_ = *E*_*A*_(1 − *M*_*A*_)*M*_*I*_
Biomass production	*M*_*I*_ + *k*_*B*_*M*_*A*_ + *E*_*B*_ → *M*_*B*_ + *k*_*B*_(1 − *M*_*A*_) + *E*_*B*_ *v*_3_ = *E*_*B*_*M*_*A*_*M*_*I*_
Storage production	$M_{I}+k_{S_{+}}M_{A}+E_{S_{+}}\to M_{S}+k_{S_{+}}(1-M_{A})+E_{S_{+}}$ *v*_4_ = *E*_*S*+_*M*_*A*_*M*_*I*_
Storage degradation	$M_{S}+k_{S_{-}}M_{A}+E_{S_{-}}\to M_{I}+k_{S_{-}}(1-M_{A})+E_{S_{-}}$ *v*_5_ = *E*_*S*−_*M*_*A*_*M*_*S*_
Maintenance reaction	*M*_*A*_ → (1 − *M*_*A*_) *v*_6_ = *K*_0_ + *K*_*S*_*M*_*S*_ + *K*_*E*_∑_*i* ≠ *T*_ *E*_*i*_
Parameters	*k*_*A*_ = 30; *k*_*B*_ = 5; *k*_*T*_ = 1; $k_{S_{+}}\;=\;4$; $k_{S_{-}}\;=\;1$ *K*_0_ = 1; *N*_0_ = 1; *K*_*S*_ = 0.01; *K*_*E*_ = 1; *E*_*T*_ = 0.5
Rate equations	${\dot{M}}_{A}=-k_{T}\;v_{1}+k_{A}\;v_{2}-k_{B}\;v_{3}-k_{S+}\;v_{4}-k_{S-}\;v_{5}-v_{6}$ ${\dot{M}}_{I}=v_{1}-v_{2}-v_{3}+v_{5}-v_{4}$; ${\dot{M}}_{S}=v_{4}-v_{5}$; ${\dot{M}}_{B}=v_{3}$

Reaction rates are based on first-order rate laws, except the zero-order maintenance reaction rate. * indicates that the reversible reaction occurs only for *N* > *M*_*I*_ with an Heavyside function H in the rate law. 1 − *M*_*A*_ denotes the converted form of *M*_*A*_ with a unit total pool concentration. Note that *E*_*i* = *A*, *B*, *S*+/−_ (not *E*_*T*_) are time-dependent variables given by Eq 6 or Eqs 7 and 8. Dimensions: [*M*_*i*_] = *C*, [*E*_*i*_] = *C*^−1^ *T*^−1^, [*v*_*i*_, *K*_0_] = *CT*^−1^, [*K*_*s*_] = *T*^−1^ and [*K*_*E*_] = *C*^2^. Normalized units: the unit of concentrations *C* is defined by the total pool of ATP and ADP (that is about 10 mM) and the unit of time *T* is determined by the unit value of the basal decay rate of ATP *K*_0_ and would typically correspond to 10 s.

The time evolution of metabolic variables follows the differential equation system:

$$\frac{d\vec{M}}{dt}=\vec{F}\left(\vec{M}\left(t\right),\vec{E}\left(t\right),N\left(t\right),K\left(t\right)\right)$$ (1)

where $\vec{F}$ is given in Table 1. The rate laws written in Table 1 assume that biochemical reactions are not elementary chemical reactions but the result of multistep or composite reactions. For instance, the production of *k*_*A*_ = 30 ATP from *k*_*A*_ ADP and one *M*_*I*_ is the net result of multiple reactions within the Krebs cycle and the electron transport chain, rather than the result of a single 31-body collision reaction. Therefore, reaction rate exponents are not derived from the stochiometric coefficients as in mass-action law (*r* ∝ *M*_*I*_ (1 − *M*_*A*_)^30^), but are assumed to be of the lowest first order for each substrate (*r* ∝ *M*_*I*_ (1 − *M*_*A*_)) as it would be the case for a linear chain of *k*_*A*_ reaction producing one ATP each. Besides, it is also to mention that two reactions are assumed to be catalyzed by constant enzyme level. First, the nutrient transport reaction rate (*v*_1_ in Table 1) is described by a non-regulated anisotropic diffusion process. Considering *E*_*T*_ as a parameter amounts to consider that the nutrient uptake rate has an upper bound of *E*_*T*_ *N*_0_ and is mainly driven by extracellular nutrient concentration levels, allowing us to focus mainly on the dynamic regulation of anabolic versus catabolic processes. Second, the maintenance (i.e., housekeeping) reaction is described as a zero-order reaction for ATP and depends on the concentration of storage and enzyme (*v*_6_ in Table 1). The zero-order reaction rate reflects the conservation of the metabolic flux dedicated for maintenance regardless the variations of substrate (e.g., ADP) concentrations.

The sources of non-stationarity in the model are of two sorts: the changes in extracellular nutrient levels *N*(*t*) and the changes in energy demand *K*(*t*) as ATP-consuming cellular functions (stress management, motility, morphological changes…) are prone to be sensitive to environmental changes and transient in time. For simplicity, we consider sinusoidal variations of *N*(*t*) and *K*(*t*):

$$N\left(t\right)=N_{0}-a_{N}\frac{1-\text{cos}\left(\omega t\right)}{2}$$ (2)

$$K\left(t\right)=K_{0}+a_{K}\frac{1-\text{cos}\left(\omega t\right)}{2}$$ (3)

where *a*_*N*,*K*_ are the perturbation amplitudes from such basal levels *N*_0_ and *K*_0_ and *ω* is the perturbation angular frequencies (hereafter referred to as perturbation frequency). Note that *a*_*N*_ varies between 0 and *N*_0_ to satisfy *N* ≥ 0 for all time *t*. Given these non-stationary conditions, the optimization criterion for metabolic fitness is the time-averaged biomass production rate (called thereafter growth rate) in the permanent regime:

$$\begin{array}{cc} \Phi & =\frac{1}{T}\int_{t_{0}}^{t_{0}+T}E_{B}\left(t\right)M_{I}\left(t\right)M_{A}\left(t\right)dt\\ M_{i}\left(t_{0}+T\right) & =M_{i}\left(t_{0}\right),\;\;\;\left\{i=I,A\right\} \end{array}$$ (4)

where [*t*_0_, *t*_0_ + *T*] is the sampling time window and *T* = 2*π*/*ω*.

### Metabolic parameter values

The prototypical model of metabolism depicted in Fig 1 is not specific to a particular organism and does not take into account the diversity of nutrient sources, storage compounds and functional biomass compounds (e.g., DNA, RNA, proteins…). As a result, model parameter values are not necessarily related with known reaction rates and stochiometries associated with a selected metabolic pathway. Nevertheless, the choice of parameter values (Table 1) has been made to match the order of magnitudes of some global or averaged biological quantities, such as the ATP concentration and lifetime, the glucose uptake rate or the glucose-dependent ATP production. Parameter values are dependent on the concentration and time unit chosen. The assumption that the total concentration of the pool of ATP and ADP is constant and equal to 1 defines the unit of concentration that is set to 10 mM as the experimentally measured value for ATP concentration is typically between 1 mM to 10 mM depending on the type and state of the cell [33]. The unit of time is given by the choice that the basal decay rate of ATP is unit-normalized with *K*_0_ = 1. The biological value of the basal consumption rate of ATP can be approximately derived from the respiratory rates *J*_*ATP*_ ∼ 50 mM.min^−1^ measured in yeast cells in the stationary phase [34]. Given the concentration unit defined above and an ATP:ADP ratio of about ∼5: 1 [33], the consumption rate would be 6 min^−1^ which corresponds to a time unit of 10 s. This upper bound of ATP lifetime is consistent with the measured values of ATP turnover time of the magnitude of second in diverse growth conditions and species [33].

Parameters for ATP production and consumption are chosen from the global gain or cost of ATP associated with a whole metabolic process. The value of ATP gain associated with TCA cycle is set to *k*_*A*_ = 30, which is similar to the order of magnitude of 25 g of ATP produced through the oxidation of 1 g of acetyl COA (both metabolites have a similar molar mass). The value of ATP consumption associated with biomass production is set to *k*_*B*_ = 5 as the minimal energy-cost for protein synthesis is 5 ATP hydrolyzed for each peptide bond formed, assuming that the molar mass of peptide is similar to that of ATP and neglecting other biosynthetic costs. The ATP cost for the whole process of storage production, maintenance and consumption depends on the type of storage compounds. We use the following arbitrary values $k_{S_{+}}\;=\;4$, $k_{S_{-}}\;=\;1$ *K*_*S*_ = 0.01 and have checked a posteriori that storage content is lower than 10 times the adenyl phosphate content *M*_*s*_ < 10, as starch or glycogen contents is usually limited to a maximum of a few percent of cell mass, whereas ATP content of the magnitude of 0.1%. We have also made a careful sensitivity analysis of these parameters (see Supplementary Material). The parameter value *E*_*T*_ for nutrient import is based on the glucose import rate measured in budding yeast. Depending on the extracellular glucose concentration and the type of hexose transporter involved, glucose import rate can be estimated between 10 and 100 min^−1^ [35], which translates into 0.5 < *E*_*T*_ < 5 for the units defined above of 10 mM for concentrations and 10 s for time. The parameter choices *E*_*T*_ = 0.5 and *N*_0_ = 1 correspond to a maximal glucose uptake rate of *E*_*T*_ *N*_0_ = 0.5 mM.s^−1^ (as *M*_*A*_ < 1 and (*N*(*t*) − *M*_*I*_) < *N*_0_), which is consistent with the maximal glucose uptake rate measured for yeast cells of 210^7^ molecules per second that gives approximately 1 mM.s^−1^ [36] for a yeast cell diameter of 5 *μ*M.

### Parameter optimization using perturbation method

The search for regulatory parameters that shape $\vec{E}\left(t\right)$, so as to maximize the growth rate Φ in dynamic environments requires to use parameter optimization techniques. Perturbation methods are well adapted in the case of small enough environmental fluctuations. Environmental, enzyme and metabolic variables can be expanded up to first order *x* = *x*_0_ + *ϵx*_1_ (*x* = *M*_*i*_, *E*_*i*_, *N*, *K*) where first-order terms are real trigonometric polynomial functions of the phase *θ* = *ωt*:

$$x_{1}=c_{i}+\sum_{i=1}^{n}a_{i}\;\cos\left(i\theta \right)+b_{i}\;\sin\left(i\theta \right)$$ (5)

where *n* = 1 for *x* = *E*_*i*_, *N*, *K* and otherwise undefined. By substituting Eq 5 into Eqs 1 and 4, the vector field $\vec{F}$ and the growth rate Φ are expanded in power series of *ε*, leading to a hierarchy of equations for $\dot{\vec{M}}$ and Φ that can be solved recursively by using a formal calculus software (Maple). To the 0th order in *ε*, the steady state condition ${\vec{F}}_{0}({\vec{M}}_{0},{\vec{E}}_{0},{\left\{N,K\right\}}_{0})=\vec{0}$ allows ${\vec{M}}_{0}$ to be expressed as a function of ${\vec{E}}_{0}$ and to be substituted into Φ_0_. Optimal enzyme parameters ${\vec{E}}_{0}$ are obtained by finding the single local maximum of Φ_0_ that satisfies $\nabla \Phi_{0}\left({\vec{E}}_{0}\right)=0$ and $\nabla^{2}\Phi_{0}\left({\vec{E}}_{0}\right)>0$. As well, solution of the linear equations at the following *l*th orders $\dot{{\vec{M}}_{l}}={\vec{F}}_{l}$ allows to find asymptotic time-dependent solutions ${\vec{M}}_{l}\left(t\right)$ as a function of trigonometric polynomial coefficients *a*, *b* and *c* of ${\vec{E}}_{l}$, while the optimized coefficients are obtained again by maximizing Φ_*l*_.

### Parameter optimization using evolutionary algorithm

For a fluctuation of *N* or *K* of any amplitude (not necessarily small), parameter optimization is performed via a population-based metaheuristic algorithm called evolution strategy (ES) (S1 Fig) that is a specific class of evolutionary algorithm [37]. The (*n*, *n*)-ES algorithm copes with a pool of *n* real parameter vectors (the parents) whose fitness score given by the growth rate Φ is known. The optimization process contains three main steps: reproduction, mutation, selection. The reproduction step consists in generating *n* offspring from the *n* parents: *n* times, a parent is randomly selected (with uniform distribution) to be duplicated. No fitness criterion is thus applied to the reproduction step and a parent can give more than one offspring. During the mutation step, the parameters of the offspring are modified with a probability *p* through multiplication by a factor 10^*r*^ or through the addition of a term *r*, where *r* ∈ [−*fw*, *fw*] is a random number of uniform distribution: *fw* and *p* quantify the amplitude and the probability of the mutation. Specific boundary conditions may apply such as *a*_*i*_ ∈ [0, 1] or periodic boundary condition for *φ*_*i*_ associated with Eq 6. The selection step first evaluates the fitness of the *n* offspring, and then selects the *n* highest-fitness individuals in the pool of 2*n* parameter sets (parents plus offspring) to compose the parents of the next generation. The selection criterion is elitist and a parent can stay in the pool as long as its fitness allows it. Finally, the optimization process terminates after a maximal number of generations (*N*_*gen*_ = 4000) In this study, the values of optimization parameters are *n* = 10 and *p* = 1, while the mutation type (multiplicative or additive) and the mutation amplitude *fw* depend on the parameter that is optimized. The best parameter set can be further optimized via a stochastic hill climbing to check the finding of a maximum. Because the end result of an evolutionary computation is sensitive to the initialization procedure of the population, we have also used a continuation method (the best solution for *a* is reused when initializing the evolutionary computation for *a*′ > *a* + *δ*) and statistical analysis of multiple evolutionary trials.

Model simulations and evolutionary computations are performed using Fortran programming language and numerical integration of differential equation is based on the extrapolation-based SEULEX Fortran routine.

## Results

### Steady-state metabolic adaptations: ATP homeostasis and metabolic collapse

A preliminary step in studying metabolic adaptation to transient stress is first to analyse the steady-state properties of the model. In stationary condition defined by constant levels of nutrient concentration *N*_0_, energy demand *K*_0_ and enzyme-dependent rate coefficient ${\vec{E}}_{0}$, a stable metabolic state corresponds to a fixed point of Eq 1 with non-negative value of nutrients, metabolites, storage, ATP and ADP. However, for some range of values of *N*_0_, *K*_0_ and ${\vec{e}}_{0}$, a stable metabolic state may not exist, such that all phase space trajectories drift toward the region of negative ATP level (*M*_*A*_ < 0), in which case metabolic death occurs. As mentioned in Methods section, an optimal stable metabolic steady state is associated with a set of enzyme parameters ${\vec{E}}_{0}$ that maximize the growth rate Φ for any values *N*_0_ and *K*_0_.

**Fig 2:**
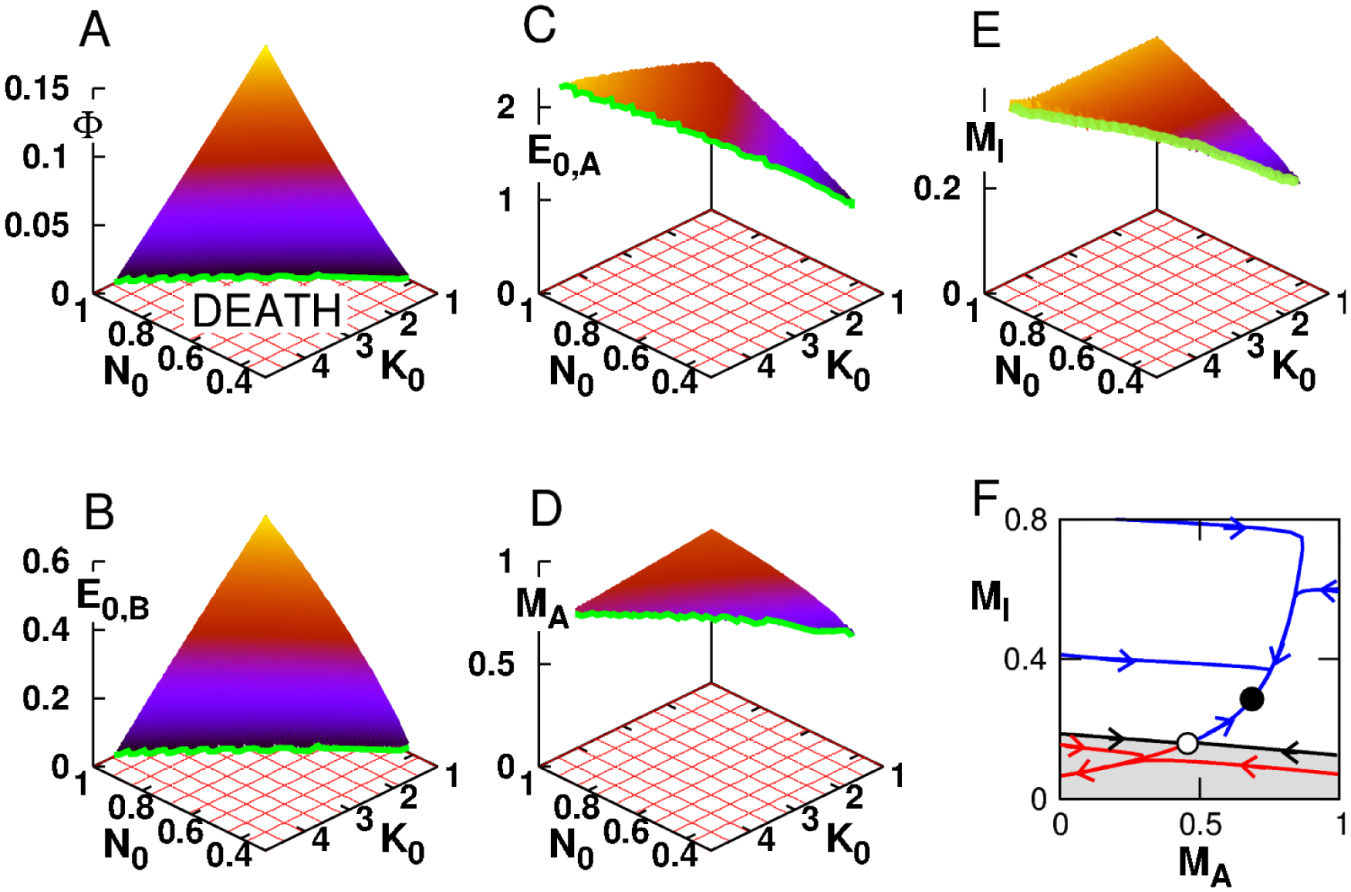
Optimal steady-state solutions. Case where rate coefficients *E*_*i*_ are optimized parameters. Properties of the optimal steady-state solution (*a*_*N*,*K*_ = 0) as a function of the stationary level of nutrient *N*_0_ and demand *K*_0_: (A) Growth rate Φ where green line corresponds to Φ = 0; (B-C) Stationary enzyme level *E*_0,*B*_ and *E*_0,*A*_; (D,E) Stable fixed point coordinate *M*_*A*_ and *M*_*I*_; (F) Example of phase space portrait and trajectories for *N*_0_ = 0.8 and *K*_0_ = 2 where black and white circles indicate stable and unstable fixed points and the black line separates the viability domain (white) and metabolic collapse domain (gray).

Fig 2 recapitulates the general properties of the optimal stable metabolic steady state. The optimal growth rate Φ decreases with decreasing values of *N*_0_ and increasing values *K*_0_ to Φ = 0 at a threshold value *N*_0,*c*_(*K*_0_) and *K*_0,*c*_(*N*_0_) (Fig 2A) beyond which metabolic death always occurs. This stress-induced decrease of Φ is paralleled with a decay of biomass production enzyme *E*_0,*B*_ to 0 (Fig 2B), while the ATP production enzyme *E*_0,*A*_ is either slightly increased or decreased depending on whether it is a nutrient stress or an energy stress, respectively (Fig 2C). In this result, the fact that the regulation of *E*_0,*A*_ is weaker than the regulation of *E*_0,*B*_ reflects the imperative need to maintain relatively constant and high levels of ATP for survival at the expense of a much reduced biomass production and flux (Fig 2D). Furthermore, the opposite regulation of *E*_0,*A*_ between the two types of stress reflects the fact that the ATP-producing and ATP-consuming reactions can be differentially affected by the two stress types, such that stress-specific and finely tuned regulation are required to reestablish ATP homeostasis. In contrast with these subtle mechanisms of ATP homeostasis, the optimal level of internal metabolites *M*_*I*_ roughly scales with external nutrients *N*_0_ (Fig 2E). Finally, an expected feature of the optimal steady state is the absence of storage enzymes $E_{0,S_{+/-}}\;=\;0$ as the processes of production, maintenance and degradation of storage generate metabolic costs in ATP and enzymes and any profits in stationary conditions. Note that even for the optimal enzyme parameters, a stable fixed point coexists with a saddle fixed point (Fig 2F), such that transition to death can arise at the threshold values of *K*_0,*c*_ and *N*_0,*c*_ through a saddle-node bifurcation but also through transient perturbations. Quantitatively, the value Φ ∼ 0.12 obtained for *N*_0_ = 1 and the nutrient threshold of *N*_0_ = 0.3 corresponds to a doubling time of ∼ 10 h for an external glucose concentration of 25 mM, which matches the order of magnitude of experimental values [38].

In sum, the steady-state and bifurcation analysis of the model allowed to identify two properties that will be key for the further study of dynamic response to transient stress: (i) the existence of a dynamic instability (saddle-node bifurcation) that corresponds to metabolic collapse and death; (ii) the fact that steady-state adaptation can be different depending on whether the stress is primarily cause by lower nutrient in-flow or a higher metabolic demand.

### Dynamic metabolic adaptations: just-in-time and storage strategies

**Fig 3:**
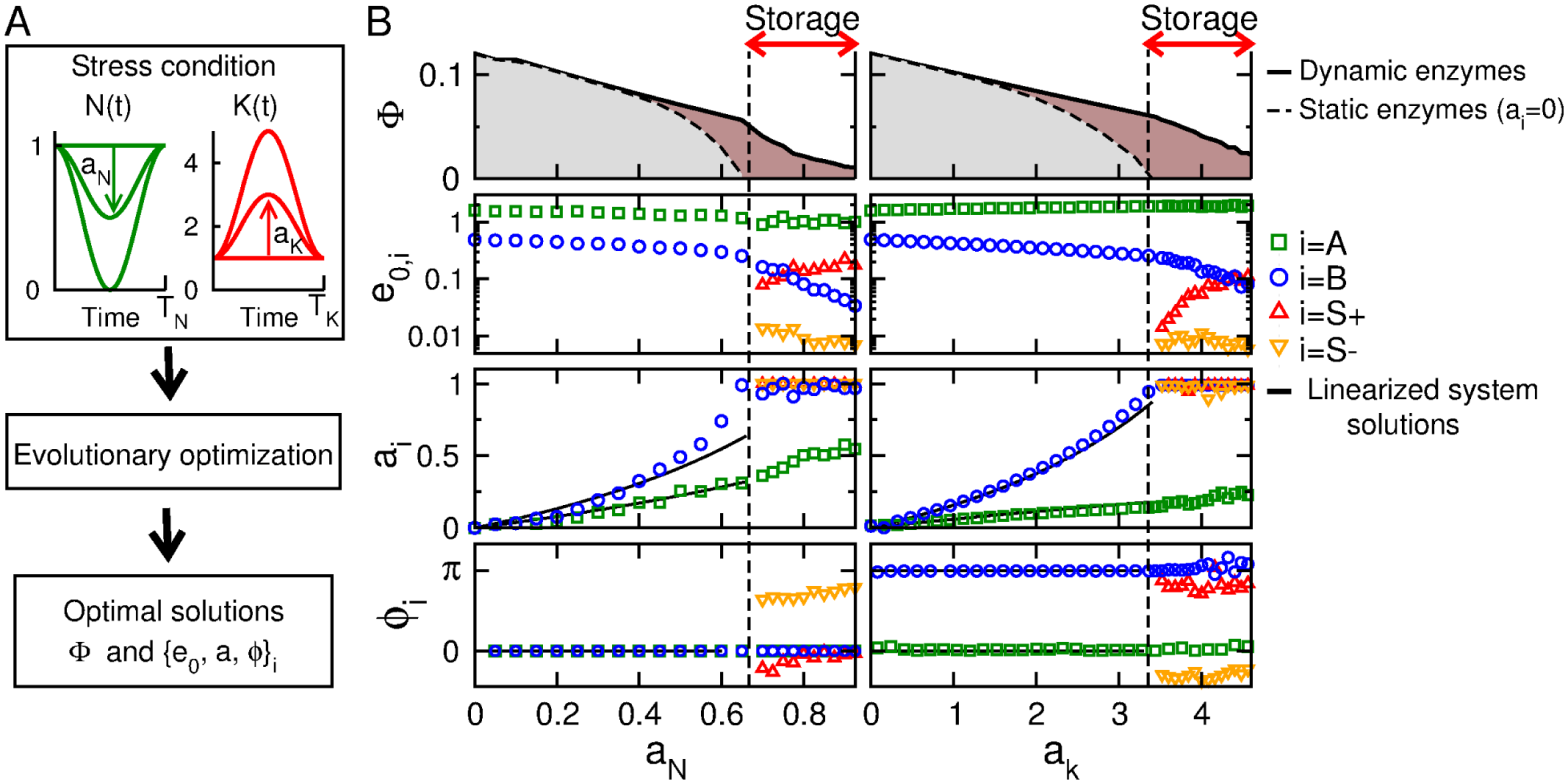
Optimal enzyme profile in the presence of time-dependent nutrient or energy stress. Case where rate coefficients *E*_*i*_(*t*) have optimized oscillatory time courses (Eq 6). (A) Simulation protocol to obtain optimal enzyme profiles as a function of the stress condition. (B) Optimal solutions as a function of stress type and amplitude reveal the existence of storage-based solutions for large enough amplitude. Upper panel: Growth rate Φ, mean enzyme levels *e*_0,*i*_, amplitude of enzyme oscillations *a*_*i*_, phase of enzyme oscillations *φ*_*i*_. Optimal enzyme profiles obtained with evolution methods (colored points) are compared with small amplitude solutions obtained with perturbation methods (continuous lines).

After having characterized the main features of the optimal metabolic steady state, the following step is to search for optimal enzyme profiles in response to non-stationary environmental conditions such as oscillations of *N*(*t*) and *K*(*t*) of amplitudes *a*_*N*,*K*_ given by Eq 2 (Fig 3A).

For simplicity, we assume a sinusoidal shape for $\vec{E}\left(t\right)$

$$E_{i}\left(t\right)=e_{0,i}\left(1+a_{i}\cos\left(\omega t+\varphi_{i}\right)\right)$$ (6)

The optimal values for the means *e*_0,*i*_, the amplitudes *a*_*i*_ and the phases *φ*_*i*_ can first be derived for small amplitude oscillations *a*_*i*_ by using a perturbation method, and then obtained for any perturbation amplitude by using evolutionary methods (see Methods section). The results depicted in Fig 3B shows that the optimal solutions are well predicted by the perturbation approach, up to relatively large stress amplitudes *a*_*N*,*K*_, which can be explained by the almost linear relation between the biomass production flux Φ and the parameter perturbation (Fig 2A). For these optimal solutions, the oscillations of *E*_*i*_ display an increasing amplitude with perturbation amplitude and are in phase or antiphase with the perturbation depending on whether *de*_0,*i*_/*dN* and *de*_0,*i*_/*dK* are positive or negative in (Fig 2B and 2C). The in-phase or anti-phase relationship between enzyme and perturbation oscillations is related with the assumption of a low perturbation frequency *ω* = 0.01 (i.e, dimensionalized period of *T* = 100 mn) that is much smaller than the natural frequency *ω*_0_ of the metabolic system, while phase shifts would occur for *ω* ∼ *ω*_0_. However, above some critical perturbation amplitude *a*_*i*,*c*_ > *a*_*i*,*c*_ (*i* = *N*, *K*), the optimal solutions display qualitatively different properties characterized by a tight regulation of storage production and degradation (*e*_0,*S*_ > 0, *a*_*S*+,*S*−_ = 1 and *φ*_*S*+_ ∼ *φ*_*S*−_+*π*). During the optimization procedure, all the enzymatic parameters converge to unique and precise values with relatively low variability. This reflects the identifiability or non-degeneracy of the model with respect to these enzymatic parameters. The means, amplitudes and phases of enzyme profiles are equally important in contributing to survival and to growth rate optimality as confirmed by the local analysis of the fitness landscape near a given optimum by measuring the variance and the correlation of enzymatic parameters (S2 Fig).

**Fig 4:**
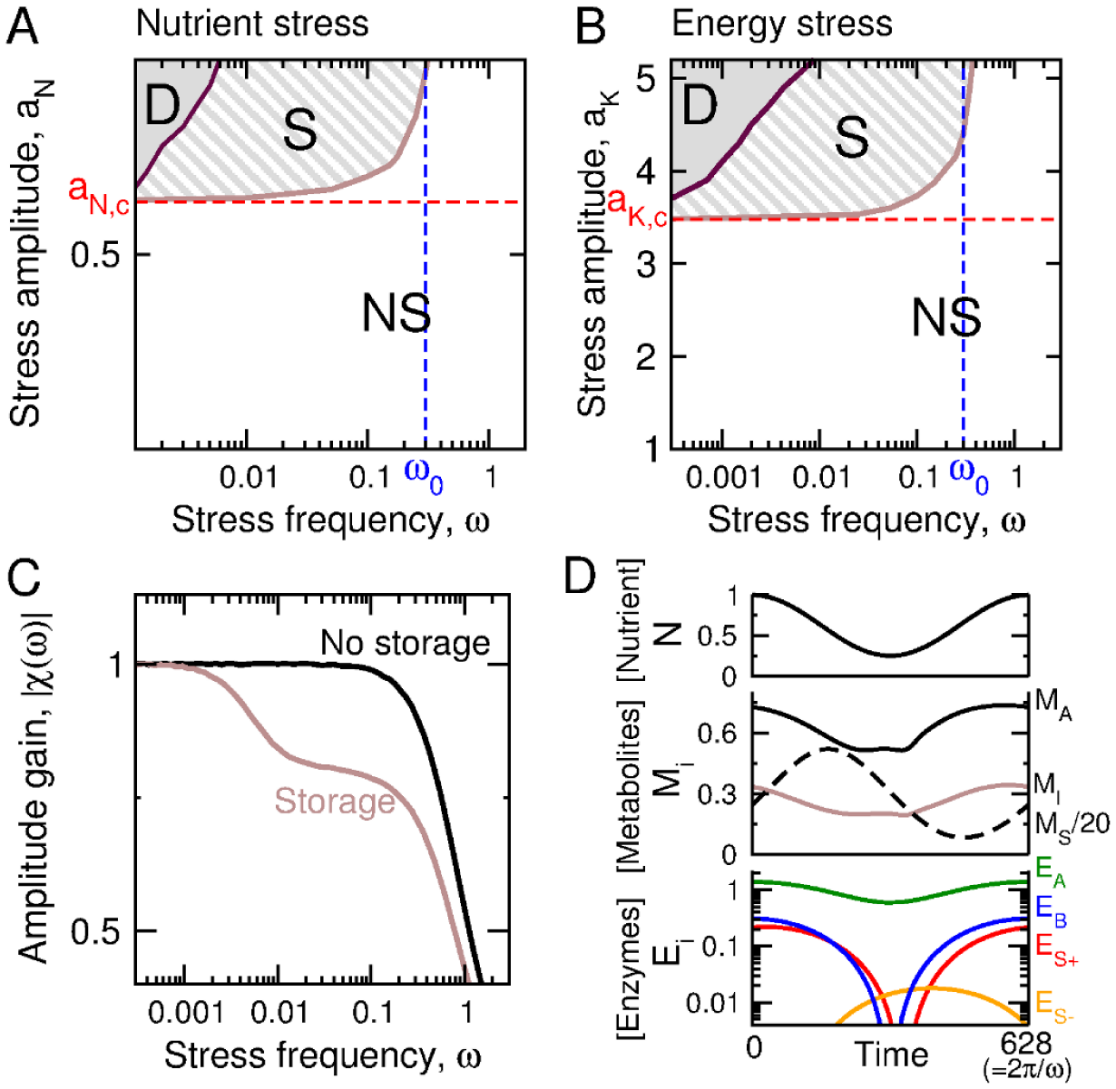
Storage strategy. Case where rate coefficients *E*_*i*_(*t*) have optimized oscillatory time courses (Eq 6). (A,B) Distinct classes of optimal solutions (NS: no storage; S: Storage; D: Death) as a function of the type, the amplitude and the frequency of the stress condition. Threshold amplitudes obtained in Fig 2, and natural frequencies are also shown. (C) Amplitude gain as a function of perturbation frequency in presence of static enzyme with and without storage reveals specific low-pass filtering properties. (D) Timecourse of metabolic and enzymatic variables close to an optimal solution associated with a nutrient stress of amplitude *a*_*N*_ = 0.75 and frequency *ω* = 0.01. S1 File provides a SBML file corresponding to this simulation.

These storage-based solutions occur for a given frequency range of oscillatory perturbations (Fig 4A and 4B). The upper-bound frequency coincides with the undamped natural (also cutoff) frequency *ω*_0_ of the low-pass second-order filter associated with the metabolic system {*M*_*A*_, *M*_*I*_} linearized around the steady state *N*_0_ = *k*_0_ = 1. The storage strategy thus confers a fitness benefit for slow variations of *N*(*t*) or *K*(*t*) below the threshold values of *N*_0,*c*_ or *K*_0,*c*_, which would not be filtered out and would induce metabolic death in the absence of slow storage cycles. In turn, the lower-bound frequency indicates that a too long stress requires to be anticipated with high storage reserves that comes with an unbearable cost and death.

The position of the boundaries that delimit the S-domain of storage-based solutions depends on model parameters (S3 Fig). For instance, the metabolic benefit of the storage strategy decreases when the costs associated with storage production ($k_{S_{+}}$), degradation ($k_{S_{-}}$) or maintenance (*K*_*S*_) increase. In that case, the value of the lower-bound frequency increases while the storage regime disapears at the expense of the death regime. In contrast, lower ATP consumption for maintenance (*K*_0_) or regulation (*K*_*E*_) or higher ATP production (*k*_*A*_) raises the stress amplitude threshold *a*_*N*,*c*_ for which the storage strategy confers a benefit. Finally, increasing anabolic costs (*k*_*B*_) merely reduces the biomass production. Nevertheless, the optimality of storage-dependent and -independent modes of metabolic adaptation depending on perturbation frequency and amplitude is a robust feature of the model.

The mechanism through which the accumulation and degradation of storage material buffer out slow environmental fluctuations can be captured by the low-pass filter component in presence of stationary levels of enzymes *E*_*i*_(*t*) = *E*_0,*i*_ (Fig 4C). The dominant cutoff frequency coincides with the environmental frequency for which the system has been optimized. The optimal profiles of the enzymatic variables and the corresponding time courses for metabolic variables depicted in Fig 4D illustrate how the optimal solution coincides with a temporal management of storage material, in order to ensure that *M*_*A*_ and *M*_*I*_ remain in the viability domain (shown in Fig 2F), so as to avoid metabolic collapse.

In all, the results show that the optimal oscillatory profiles of enzymes *E*_*j*_ tightly depends on the nature and the amplitude of the non-stationary conditions and emphasizes that a specific storage strategy appears for severe stress condition in terms of ampliitude and frequency, while the control of catabolism is slightly different depending on whether the stress corresponds to a lack of external nutrient or a strong energy requirement for homeostasis.

### Optimal signaling and regulation for dynamic adaptations to metabolic stress

The optimal oscillatory profiles of *E*_*j*_ obtained for various non-stationary conditions provide guidance on the signaling and regulatory architecture, that is the manner how these enzymes would be optimally regulated by specific signaling cues. For instance, the optimal phases of enzyme oscillations with respect to signal oscillations (see low panels of Fig 3) are expected to predict whether these enzymes would be positively or negatively regulated by the signaling pathways sensitive to these signals. It remains, however, difficult to foresee which signaling cues and how many signaling pathways are required to regulate metabolism in an optimal manner.

To address these issues, the metabolic network model given by Eq 1 is supplemented by a minimal description of the signaling pathways that regulate the time evolution of enzyme-dependent rate coefficients:

$$\tau_{i}\frac{dE_{i}}{dt}=\mu_{i}\prod_{j=1}^{N_{Y}}f_{ij}\left(Y_{j}\right)-E_{i}$$ (7)

where *μ*_*i*_ is the basal activation rate of *E*_*i*_, *τ*_*i*_ is the inactivation or degradation timescale of the enzyme (*τ*_*i*_ determines the timescale of enzymatic changes), *Y*_*j*_ is the signal input that can depend on any environmental or metabolic variables, and *N*_*Y*_ is the number of signaling pathways. The function *f*_*ij*_ is described by:

$$f_{ij}\left(x\right)=\frac{1+\lambda_{ij}{\left(\frac{x}{\theta_{ij}}\right)}^{n_{H}}}{1+{\left(\frac{x}{\theta_{ij}}\right)}^{n_{H}}}$$ (8)

which can be casted into a constant term (basal transcription) and a Hill function (regulated transcription). *λ*_*ij*_ > 1 and *λ*_*ij*_ < 1 correspond respectively to the *Y*_*j*_-dependent activation and inactivation rate of *E*_*i*_ by *Y*_*j*_. *θ*_*ij*_ is the regulatory threshold (the inflection point of the response curve for *n*_*H*_ = 2), and *n*_*H*_ is the Hill coefficient or slope factor that is set to 2, which is the minimal value that can be used to described the sigmoidal behavior of transcription kinetics. Such nonlinear behavior can arise through the affinity, cooperativity, or multimerization of transcription factors at their binding sites within target gene promoters.

**Fig 5:**
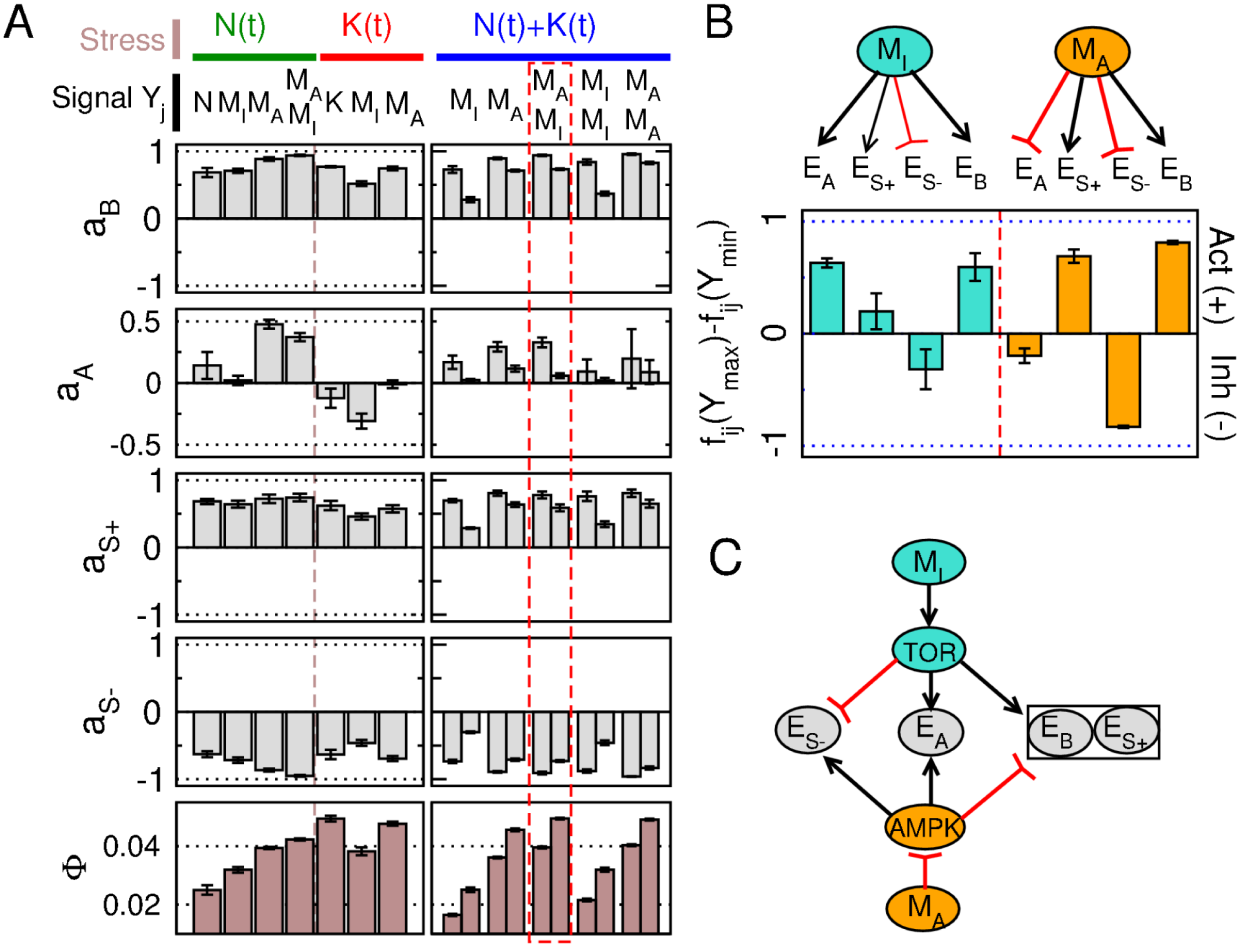
Optimal signaling and regulatory pattern of enzymes in response to single or combined stress conditions. Case where rate coefficients *E*_*i*_(*t*) are regulated by optimized signaling pathways (Eqs 7 and 8). (A) Enzyme amplitudes (Eq 9): average and variance values computed for the 20 best solutions overs 40 evolutionary runs. Evolutionary optimization is made on the regulatory parameters *μ*_*i*_, *λ*_*ij*_ and *θ*_*ij*_ for two distinct and combined stress conditions (*N*(*t*): *N*_0_ = 1, *ω* = 0.01, *a*_*N*_ = 0.7, *a*_*K*_ = 0; *K*(*t*): *N*_0_ = 1, *ω* = 0.01, *a*_*N*_ = 0., *a*_*K*_ = 3.5) and for various signals. For optimization in combined stress conditions (right panel), left versus right bars corresponds to enzyme amplitudes measured when exposed to a single stress, *N*(*t*) (left) or *K*(*t*) (right). (B) Optimal regulatory parameters *f*_*ij*_(*Y*_*max*_) − *f*_*ij*_(*Y*_*min*_) represented as activatory or inhibitory regulations in the presence of two signaling pathways and combined stress conditions (see dashed rectangle of (A)). (C) Corresponding regulatory scheme by assuming the existence of AMPK-like and TOR-like regulatory proteins.

Evolutionary optimization technique is applied to determine the regulatory parameters *μ*_*i*_, *λ*_*ij*_ and *θ*_*ij*_ that maximizes the flux. To begin with, we use constant value of the regulatory timescale *τ*_*i*_ = 1, which would correspond to a rapid mode of regulation (e.g, allosteric or post-translational), since, anyway, optimization leads to minimize *τ*_*i*_ in the absence of synthesis and degradation costs for enzymes. To compare the optimal solutions obtained in the cases of oscillatory versus regulated enzymes, a generic quantity for enzyme amplitude is defined as,

$$a_{i}=\cos\left(\varphi \right)\frac{E_{i}^{M}-E_{i}^{m}}{E_{i}^{M}+E_{i}^{m}}$$ (9)

where $\varphi =2\pi (t_{E_{i}}^{\prime }-t_{K,N}^{\prime })/T$ corresponds to the phase of the enzyme response ($t_{x}^{\prime }$ is the time of the maximum of *x*(*t*)). Optimization is first performed in the presence of a single stress conditions (*N*(*t*) or *K*(*t*) with *ω* = 0.01) and a single signaling pathway *N*_*Y*_ = 1 where *Y*_1_ is a function of *N*, *K*, *M*_*I*_ or *M*_*A*_ (Fig 5A, left panels). The sensing functions are *Y*(*N*) = *N*, *Y*(*M*_*I*_) = *M*_*I*_, *Y*(*K*) = 5 − *K* and *Y*(*M*_*A*_) = 0.1*M*_*A*_/(1 − *M*_*A*_) and have been chosen to display similar maximal and minimal values for *Y* given the stress intensities considered here.

Irrespective to the signaling cue, a stress associated with low levels of *N*, *M*_*I*_ or *M*_*A*_ or high levels of *K* induces an inhibition of enzymes $E_{S_{+}}$, *E*_*B*_ and activation of $E_{S_{-}}$, while it induces either an activation or an inactivation of *E*_*A*_ depending on whether it is an energy or a nutrient stress, respectively (Fig 5A, left panels). These tendencies are consistent with the optimal oscillatory pattern of enzymes (Fig 3). However, both the strengths of regulation and the growth rate Φ slightly depend on the type of signaling cues, which presumably reflects differences in the periodic time profile of *Y*_*j*_(*t*) which can be more or less sinusoidal or distorted. The result that the optimal growth rate Φ systematically augments by increasing the hill coefficient *n*_*H*_ (result not shown) or by increasing the number of signaling pathways *N*_*Y*_ is consistent with the notion that signaling complexity improves metabolic fitness through refined control of enzyme time courses in the absence of costs associated with increases of *N*_*Y*_ or *n*_*H*_.

In the case where a single signaling pathway is optimized to maximize the sum of the flux for the two stress conditions (Fig 5A, right panels), the growth rate is decreased from 10% (for *M*_*A*_) to 40% (for *M*_*I*_) compared to the case where optimization is done for each stress condition separately. This result reflects the property that optimal regulation of the ATP-production enzyme *E*_*A*_ depends on the stress type, giving rise to a compromise solution of intermediate regulation of *E*_*A*_. In contrast, optimization with two signaling pathways allows to recover the optimal fluxes obtained for each stress condition optimized separately with a single signaling pathway. This dual signaling and regulatory scheme shows a clear divisions of the sensing and regulatory task as the signaling sensitive to intermediate metabolites *M*_*I*_ inhibits the ATP-production enzyme *E*_*A*_ while the pathways sensitive to *M*_*A*_ (ATP) activates *E*_*A*_ (Fig 5B), which is reminiscent to the acknowledged pattern of regulation by AMPK and TOR (Compare Figs 5C and 1A). Besides their opposite regulation of *E*_*A*_, the two signaling pathways also differ in the regulatory strength of storage enzyme, which also suggests a division of tasks based on the survival-growth dichotomy. The *M*_*A*_-sensitive pathway is prone to lead to drastic metabolic adaptation upon severe stress, while the *M*_*I*_-signaling pathway would rather achieve a more graded response to optimize the metabolic growth rate.

**Fig 6:**
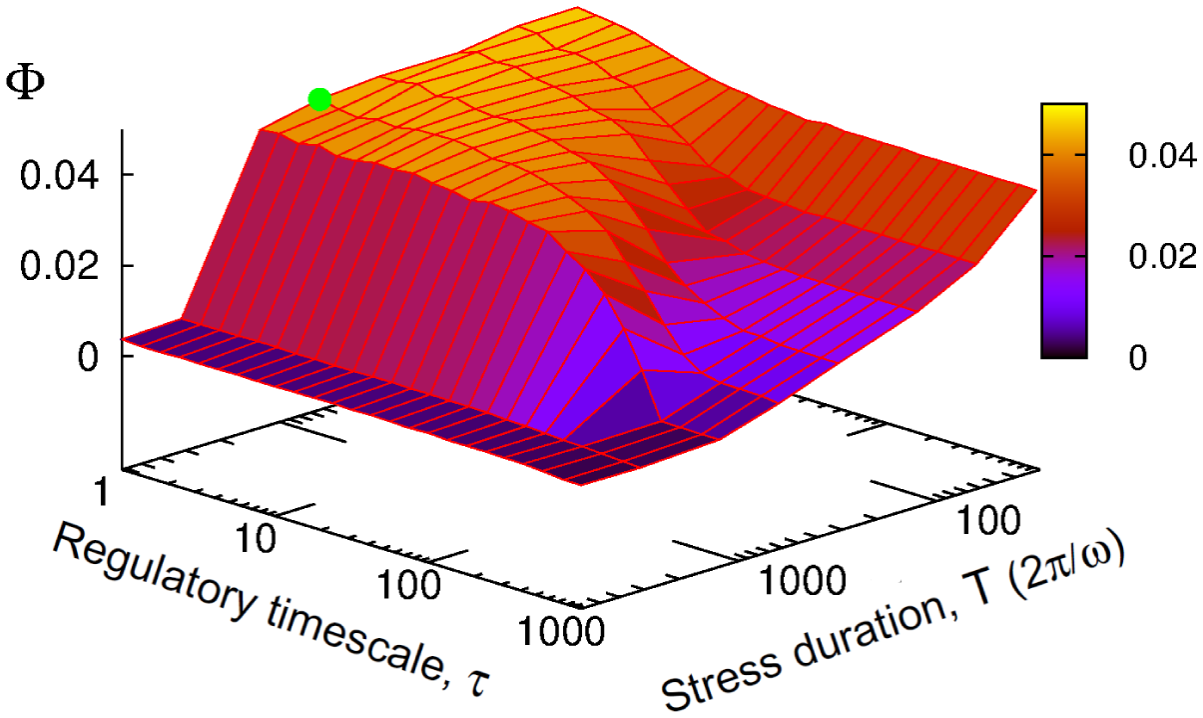
Optimal regulatory timescale. Case where rate coefficients *E*_*i*_(*t*) are regulated by optimized signaling pathways (Eqs 7 and 8). Optimal score obtained from evolutionary computation of regulatory parameters (*μ*_*i*_, *λ*_*ij*_ and *θ*_*ij*_) for a given stress and signaling condition (*a*_*N*_ = 0.6, *a*_*K*_ = 0 and *Y* = *M*_*I*_) as a function of the regulatory timescale *τ*_*i*_ = *τ* and the perturbation timescale *T* = 2*π*/*ω*. The green point indicates the values of *τ* and *T* used in Fig 5.

Although evolutionary optimization of regulatory parameters have been performed for specific values of regulatory timescale (*τ*_*i*_ = *τ* = 1) and stress timescale (*ω* = 0.01), the optimal regulatory schemes that have been obtained in various stress conditions are expected to weakly depend on *τ* as long as it is short enough compared to the period of stress oscillation *T* = 2*π*/*ω*. Fig 6 shows indeed that the optimal solution characterized by a strong regulation of storage production and degradation enzymes *E*_*S*+/−_ and biomass production enzyme *E*_*B*_ remains unchanged for a large range of *τ* as long as *τ* < *T*. For *τ* of a same or larger magnitude than *T*, the score of the optimal solution decreases reflecting the absence of temporal regulation of *E*_*i*_ due to a low-pass filtering effect of regulatory dynamics.

To summarize, the crosstalk between several signaling and regulatory pathways confers fitness advantages by refining the time profile of respective enzymes, but also by allowing a distribution of tasks when coping with different stress types and intensities.

## Discussion

We have developed a kinetic model of a prototypical regulated cell metabolism under dynamic and far from equilibrium conditions, for instance when exposed to transient and severe stress. The study case of oscillatory perturbations and the use of a coarse-grained description of metabolic and regulatory pathways are particularly convenient to study optimal regulatory patterns in non-stationary conditions [31], to relate metabolic optimality with linear and nonlinear response to frequency- and amplitude-specific perturbations [39] and to provide testable experimental predictions in terms of cut-off frequencies, time scale hierarchies and threshold amplitudes [40]. In turn, the model idealizations would preclude the possibility to make quantitative comparisons regarding flux and threshold values across diverse species or environmental conditions.

Nevertheless, the analysis of such coarse-grained model of cell metabolism could identify and analyze distinct adaptive strategies in changing environments, depending on the nature, the amplitude, and the timescale of environmental changes. In line with previous studies, adaptation to small environmental fluctuations only requires to be compensated in time by dynamically reallocating the enzyme resources [29, 41] by analogy with just-in-time manufacturing strategies [18]. In contrast, metabolic adaptation to large environmental fluctuations involves storage management pathways in order to buffer out these fluctuations and to protect cells against detrimental outcomes for survival. The buffer effect relies on a slow storage degradation process, providing a low-pass filtering property to the metabolic system. In this process, a tight regulation of the storage production and degradation is of critical importance to minimize the cost of production and of maintenance of storage material. A typical example of such adaptive mechanism is the regulation of starch, a major form of stored carbohydrates in plants: starch is accumulated during the day and remobilized at night at a rate which depends on the night length to support continued respiration [42]. In fact, different storage compounds may exhibit differential capacities in coping with rapid or slow changes of their environment, depending on the energetic and temporal constraints associated with their production, transport, reactivity, and degradation. Carbohydrates, for instance, are energy stores less concentrated than triacylglycerols, but are more rapidly mobilized. The specific roles of glycogen and trehalose during the diauxic shift response and the quiescence starvation response in yeast further suggest the existence of distinct and combined storage strategies depending on the mode of production and reactivity of storage compounds [43]. Finally, proteins and other macromolecular complexes also constitute large reserves of recyclable material that can be catabolized through the process of autophagy [44]. This diversity of catabolic processes leaves open the question of their coordination to resupply the biosynthetic precursors or the energetic compounds and to optimize survival at various timescales.

Optimal metabolic fitness in fluctuating environments requires a time-dependent regulation of storage material, but also of biosynthetic ATP-consuming processes and catabolic ATP-generating processes. While the biosynthetic machinery is switched off in any stress condition, the regulation of the ATP production through the TCA cycle is more subtle and is prone to depend on the nature of the stress. As a result, optimal regulation in various stress conditions tend to require a crosstalk between specialized signaling pathways that have both cooperative or opposing actions on selected enzymatic targets. The obtained pattern of regulation bears close similarity with the AMPK and TOR-dependent pathways, as these pathways exert antagonistic roles for storage management, autophagy, and biosynthesis, whereas both activate some other pathways such as glycolysis and mitochondrial activity. However, it remains debatable whether regulation should be mediated through post-translational or transcriptional mechanisms, given that transcription-dependent or degradation-dependent changes of expression can be too slow to track environmental changes [40, 41], while rapid protein turnover can be energetically costly. Although the optimal regulatory profile of enzymes exhibited a clear and consistent pattern, the issue of optimal sensing cues remains more difficult to apprehend. An external perturbation is propagated simultaneously through both the metabolic and signaling network in a complex manner as different perturbation modes can be either amplified or attenuated in time. On the one hand, external perturbations seem to provide more reliable cues. On the other hand, internal sensing provides information about the metabolic state, regarding how well-balanced are the respective fluxes [16], or how close a system is far from steady state or from the threshold beyond which metabolic collapse occurs. Combined mechanisms of ATP homeostasis and fast ATP turnover make the level of ATP:ADP:AMP ratio very sensitive to whether the metabolic stability is threatened or compromised, and such ratios therefore constitute good indicators of stress [7].

From a single-cell perspective, a primary role of intracellular signaling is to track environmental changes, so as to adjust the cellular state accordingly. However, efficient metabolic adaptations in microbial organisms to environmental changes can also occur in the absence of signaling through bet-hedging strategy based on the relative growth and survival rates of cells within multistable and heterogeneous population [45]. In fact, which strategy is optimal and whether these strategies could be mixed depend on many cellular and environmental parameters, such as the rates of proliferation, the randomness and frequency of environmental changes, or the timescale and energetic cost of regulation [46–48], which is reliant on the organism lifestyle, prokaryote or eukaryote, unicellular or multicellular, phototroph or chemotroph. The issue of the cellular response strategy to nutrient and energy stress thus provides a promising venue for investigating the evolution of regulatory complexity.

## Supporting Information

S1 Fig Parameter optimization through evolutionary algorithm. (A) Flowchart of the evolutionary algorithm. (B) Evolution of the growth rate score and the enzymatic parameters (mean enzyme level *e*_0,*i*_, amplitude of enzyme oscillations *a*_*i*_, phase of enzyme oscillations *φ*_*i*_ and color legend as in Fig 3) as a function of the number of generation *N*_*GEN*_. The upper panel shows the best and worst growth rate score, Φ_1_ and Φ_*n*_, of the update population of *n* individuals. Left and right panels show the cases of mild and severe stress conditions associated with stress frequency *ω* = 0.01 and amplitudes *a*_*N*_ = 0.5 (left) or *a*_*N*_ = 0.7 (right) for which the evolved optimal solutions require storage metabolism or not.
(PDF)

S2 FigVariance and correlation analysis of fitness landscape parameters.(A) Schematic description of the analysis consisting in a principal component analysis (PCA) on a parameter set satisfying some fitness-based requirements. *Left panel:* The analysis is performed for the optimal solution associated with the stress condition of amplitude *a*_*N*_ = 0.8 and frequency *ω* = 0.01 (see Fig 3A for the enzymatic parameter values). This optimal solution is defined by the optimized vector ${\vec{p}}_{opt}$ corresponding to the 12 enzymatic parameters: logarithm of the means log(*e*_0,*i*_), amplitudes *a*_*i*_, phases *φ*_*i*_ with *i* = *A*, *S*_+_, *S*_−_, *B*. To determine the geometry of the fitness landscape around this optimum, we consider perturbation vector $\vec{z}$ whose coordinates are uniformly distributed random values so as to define a unit hypercube centered at zero. Parameters are varied by *p*_*i*_ = *p*_*opt*, *i*_ + *δp*_*i*_ with *δp*_*i*_ = *z*_*i*_ Δ*p*_*i*_ whereas Δ*p*_*i*_ corresponds to the maximal variations, which are set to 10% of the possible range of values for *a*_*i*_ and *φ*_*i*_ and log(2)/2 for the means *e*_0,*i*_. *Middle panel:* Among 10^5^ samples of random parameter perturbation sets $\vec{z}$, only 599 sets ${\vec{z}}_{j=1,599}$ retains a high growth rate fitness score Φ > *θ*Φ_*opt*_ with *θ* = 0.9, from which we generate a data set *X*_*θ*_ whose columns correspond to those vectors ${\vec{z}}_{j}$. *Right panel:* The last step is to perform a PCA on this fitness-dependent data set *X*, where principal components are the eigenvectors of the correlation matrix $Q={\left(\sqrt{diag(Q)}\right)}^{-1}Q{\left(\sqrt{diag(Q)}\right)}^{-1}$ and Q is the covariance matrix $Q=X_{\theta }^{T}X_{\theta }$. PCA is a standard method to reduce the dimensionality of high dimensional data sets, and PCA applied to parameter sets satisfying high fitness gives valuable informations on the geometry of the fitness landscape around the global optimum, such as the most neutral directions and the most selective directions. (B) Standard deviations of the enzymatic parameters of the dataset *X*_*θ*_ equal to the square roots of the diagonal elements of the covariance matrix $\sqrt{Q_{ii}}$. All parameter standard deviations are significantly smaller to 1 that is the edge length of the hypercube, indicating that fitness is sensitive to all parameters (with a higher sensivity to *φ*_*S*−_ for phases and *e*_0,*B*_ for means). (C) To uncouple the informations regarding the respective variances of parameter distributions shown in (B) and the correlation between different parameters, PCA is made as the eigencomposition of the correlation matrix. *Up panel:* The eigenvalue spectrum *λ*_*Q*_ of *Q*. *Bottom panel:* the contribution to each parameter (index is defined in (C)) to the two eigenvectors associated with the two highest eigenvalues *λ*_*Q*,1_ and *λ*_*Q*,2_. The large number of eigenvalues *λ*_*Q*_ of order of 1 precludes over-parameterization and guarantees parameter identifiability, while it also entails a complex fitness landscape with correlated parameters in many eigen-directions, especially for the two principal components. (D) Correlation matrix *Q* and four examples of correlation between the most correlated enzymatic parameters (red circles correspond to the original optimal parameters).(PDF)

S3 Fig Sensitivity analysis of non-optimized model parameters. (A) The analysis is performed for the optimal solution associated with the stress condition of amplitude *a*_*N*_ = 0.8 and frequency *ω* = 0.01 (see Fig 3A for the corresponding enzymatic parameter values). Growth rate score Φ and mean value of storage production enzyme *e*_0,*S*+_ of optimized solutions as a function of the non-optimized model parameters *k*_*S*+_, *k*_*S*−_, *K*_*S*_, *k*_*A*_, *K*_*E*_, *K*_0_, which are varied independently in a log or linear scales. Upper horizontal bars indicate the corresponding metabolic regime (white: no storage; hatched grey: storage, S; grey: death, D). (B) Schematic representation of the influences of the non-optimized model parameters on the size and boundaries of the storage regime in the amplitude-frequency plane.
(PDF)

S1 File SBML file. Set of equations and parameters of the biochemical network model as a System Biology Markup Language (SBML) file. The specific set of parameters corresponds to Fig 4D.
(XML)
