# Supplementary material for: Adaptive Benefits of Storage Strategy and Dual AMPK/TOR Signaling in Metabolic Stress Response
Source: PLoS One. 2016 Aug 9;11(8):e0160247. doi: 10.1371/journal.pone.0160247 (PMC4978418; doi:10.1371/journal.pone.0160247)
Supplement: S1 Fig — (A) Flowchart of the evolutionary algorithm. (B) Evolution of the growth rate score and the enzymatic parameters (mean enzyme level e0,i, amplitude of enzyme oscillations ai, phase of enzyme oscillations φi and color legend as in Fig 3) as a function of the number of generation NGEN. The upper panel shows the best and worst growth rate score, Φ1 and Φn, of the update population of n individuals. Left and right panels show the cases of mild and severe stress conditions associated with stress frequency ω = 0.01 and amplitudes aN = 0.5 (left) or aN = 0.7 (right) for which the evolved optimal solutions require storage metabolism or not. (PDF) [file pone.0160247.s001.pdf]

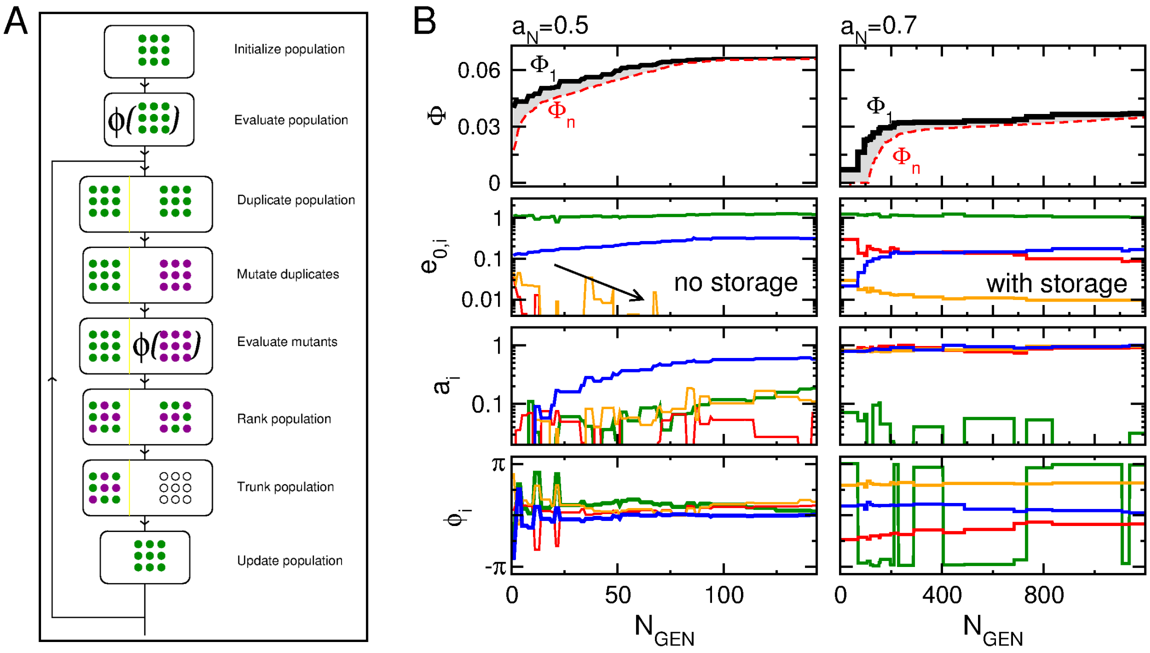

S1 Figure: Parameter optimization through evolutionary algorithm. (A) Flowchart of the evolutionary algorithm. (B) Evolution of the growth rate score and the enzymatic parameters (mean enzyme level $e_{0,i}$, amplitude of enzyme oscillations a_i, phase of enzyme oscillations φ_i and color legend as in Figure 3) as a function of the number of generation N_{GEN}. The upper panel shows the best and worst growth rate score, Φ_1 and Φ_n, of the update population of n individuals. Left and right panels show the cases of mild and severe stress conditions associated with stress frequency $\omega = 0.01$ and amplitudes $a_N = 0.5$ (left) or $a_N = 0.7$ (right) for which the evolved optimal solutions require storage metabolism or not.
