# Supplementary material for: Adaptive Benefits of Storage Strategy and Dual AMPK/TOR Signaling in Metabolic Stress Response
Source: PLoS One. 2016 Aug 9;11(8):e0160247. doi: 10.1371/journal.pone.0160247 (PMC4978418; doi:10.1371/journal.pone.0160247)
Supplement: S2 Fig — (A) Schematic description of the analysis consisting in a principal component analysis (PCA) on a parameter set satisfying some fitness-based requirements. Left panel: The analysis is performed for the optimal solution associated with the stress condition of amplitude aN = 0.8 and frequency ω = 0.01 (see Fig 3A for the enzymatic parameter values). This optimal solution is defined by the optimized vector p→opt corresponding to the 12 enzymatic parameters: logarithm of the means log(e0,i), amplitudes ai, phases φi with i = A, S+, S−, B. To determine the geometry of the fitness landscape around this optimum, we consider perturbation vector z→ whose coordinates are uniformly distributed random values so as to define a unit hypercube centered at zero. Parameters are varied by pi = popt, i + δpi with δpi = zi Δpi whereas Δpi corresponds to the maximal variations, which are set to 10% of the possible range of values for ai and φi and log(2)/2 for the means e0,i. Middle panel: Among 105 samples of random parameter perturbation sets z→, only 599 sets z→j=1,599 retains a high growth rate fitness score Φ > θΦopt with θ = 0.9, from which we generate a data set Xθ whose columns correspond to those vectors z→j. Right panel: The last step is to perform a PCA on this fitness-dependent data set X, where principal components are the eigenvectors of the correlation matrix Q=(diag(Q))-1Q(diag(Q))-1 and Q is the covariance matrix Q=XθTXθ. PCA is a standard method to reduce the dimensionality of high dimensional data sets, and PCA applied to parameter sets satisfying high fitness gives valuable informations on the geometry of the fitness landscape around the global optimum, such as the most neutral directions and the most selective directions. (B) Standard deviations of the enzymatic parameters of the dataset Xθ equal to the square roots of the diagonal elements of the covariance matrix Qii. All parameter standard deviations are significantly smaller to 1 that is the edge length of the h [file pone.0160247.s002.pdf]

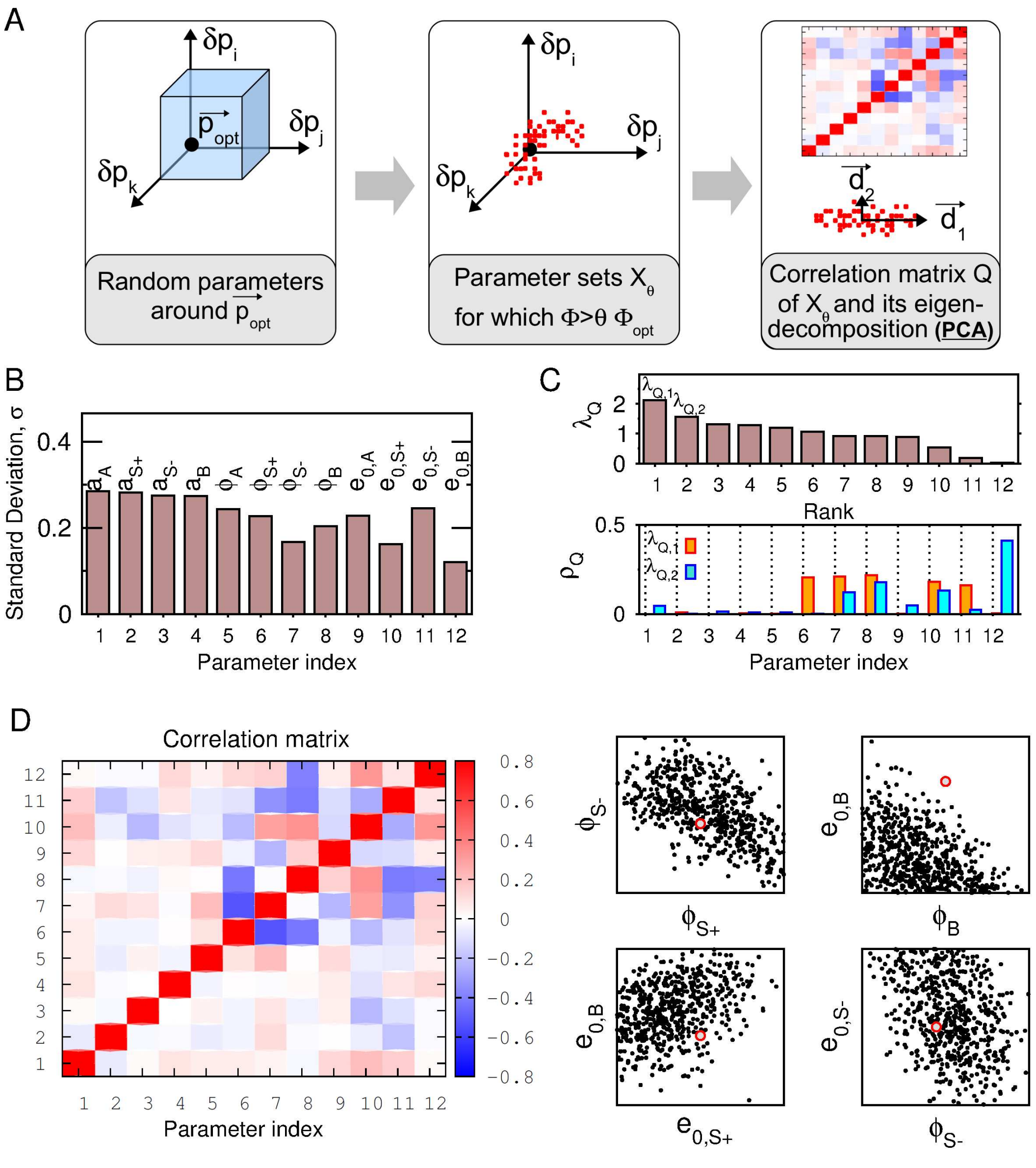

S2 Figure: Variance and correlation analysis of fitness landscape parameters.

S2 Figure: Variance and correlation analysis of fitness landscape parameters.

(A) Schematic description of the analysis consisting in a principal component analysis (PCA) on a parameter set satisfying some fitness-based requirements. *Left panel:* The analysis is performed for the optimal solution associated with the stress condition of amplitude $a_N = 0.8$ and frequency $\omega = 0.01$ (see Fig. 3A for the enzymatic parameter values). This optimal solution is defined by the optimized vector $\vec{p}_{opt}$ corresponding to the 12 enzymatic parameters: logarithm of the means $\log(e_{0,i})$, amplitudes a_i, phases φ_i with $i = A, S_+, S_-, B$. To determine the geometry of the fitness landscape around this optimum, we consider perturbation vector $\vec{z}$ whose coordinates are uniformly distributed random values so as to define a unit hypercube centered at zero. Parameters are varied by $p_i = p_{opt,i} + \delta p_i$ with $\delta p_i = z_i \Delta p_i$ whereas Δp_i corresponds to the maximal variations, which are set to 10% of the possible range of values for a_i and φ_i and $\log(2)/2$ for the means $e_{0,i}$ *Middle panel:* Among 10^5 samples of random parameter perturbation sets $\vec{z}$, only 599 sets $\vec{z}_{j=1,599}$ retains a high growth rate fitness score $\Phi > \theta\, \Phi_{opt}$ with $\theta = 0.9$, from which we generate a data set X_θ whose columns correspond to those vectors $\vec{z}_j$. *Right panel:* The last step is to perform a PCA on this fitness-dependent data set X, where principal components are the eigenvectors of the correlation matrix $Q = (\sqrt{diag(\mathcal{Q})})^{-1} \mathcal{Q} (\sqrt{diag(\mathcal{Q})})^{-1}$ and $\mathcal{Q}$ is the covariance matrix $\mathcal{Q} = X_\theta^T X_\theta$. PCA is a standard method to reduce the dimensionality of high dimensional data sets, and PCA applied to parameter sets satisfying high fitness gives valuable informations on the geometry of the fitness landscape around the global optimum, such as the most neutral directions and the most selective directions.

(B) Standard deviations of the enzymatic parameters of the dataset X_θ equal to the square roots of the diagonal elements of the covariance matrix $\sqrt{\mathcal{Q}_{ii}}$. All parameter standard deviations are significantly smaller to 1 that is the edge length of the hypercube, indicating that fitness is sensitive to all parameters (with a higher sensivity to φ_{S-} for phases and $e_{0,B}$ for means).

(C) To uncouple the informations regarding the respective variances of parameter distributions shown in (B) and the correlation between different parameters, PCA is made as the eigencomposition of the correlation matrix. *Up panel:* The eigenvalue spectrum λ_Q of Q. *Bottom panel:* the contribution to each parameter (index is defined in (C)) to the two eigenvectors associated with the two highest eigenvalues $\lambda_{Q,1}$ and $\lambda_{Q,2}$. The large number of eigenvalues λ_Q of order of 1 precludes over-parameterization and guarantees parameter identifiability, while it also entails a complex fitness landscape with correlated parameters in many eigendirections, especially for the two principal components.

(D) Correlation matrix Q and four examples of correlation between the most correlated enzymatic parameters (red circles correspond to the original optimal parameters).
