# Supplementary material for: Adaptive Benefits of Storage Strategy and Dual AMPK/TOR Signaling in Metabolic Stress Response
Source: PLoS One. 2016 Aug 9;11(8):e0160247. doi: 10.1371/journal.pone.0160247 (PMC4978418; doi:10.1371/journal.pone.0160247)
Supplement: S3 Fig — (A) The analysis is performed for the optimal solution associated with the stress condition of amplitude aN = 0.8 and frequency ω = 0.01 (see Fig 3A for the corresponding enzymatic parameter values). Growth rate score Φ and mean value of storage production enzyme e0,S+ of optimized solutions as a function of the non-optimized model parameters kS+, kS−, KS, kA, KE, K0, which are varied independently in a log or linear scales. Upper horizontal bars indicate the corresponding metabolic regime (white: no storage; hatched grey: storage, S; grey: death, D). (B) Schematic representation of the influences of the non-optimized model parameters on the size and boundaries of the storage regime in the amplitude-frequency plane. (PDF) [file pone.0160247.s003.pdf]

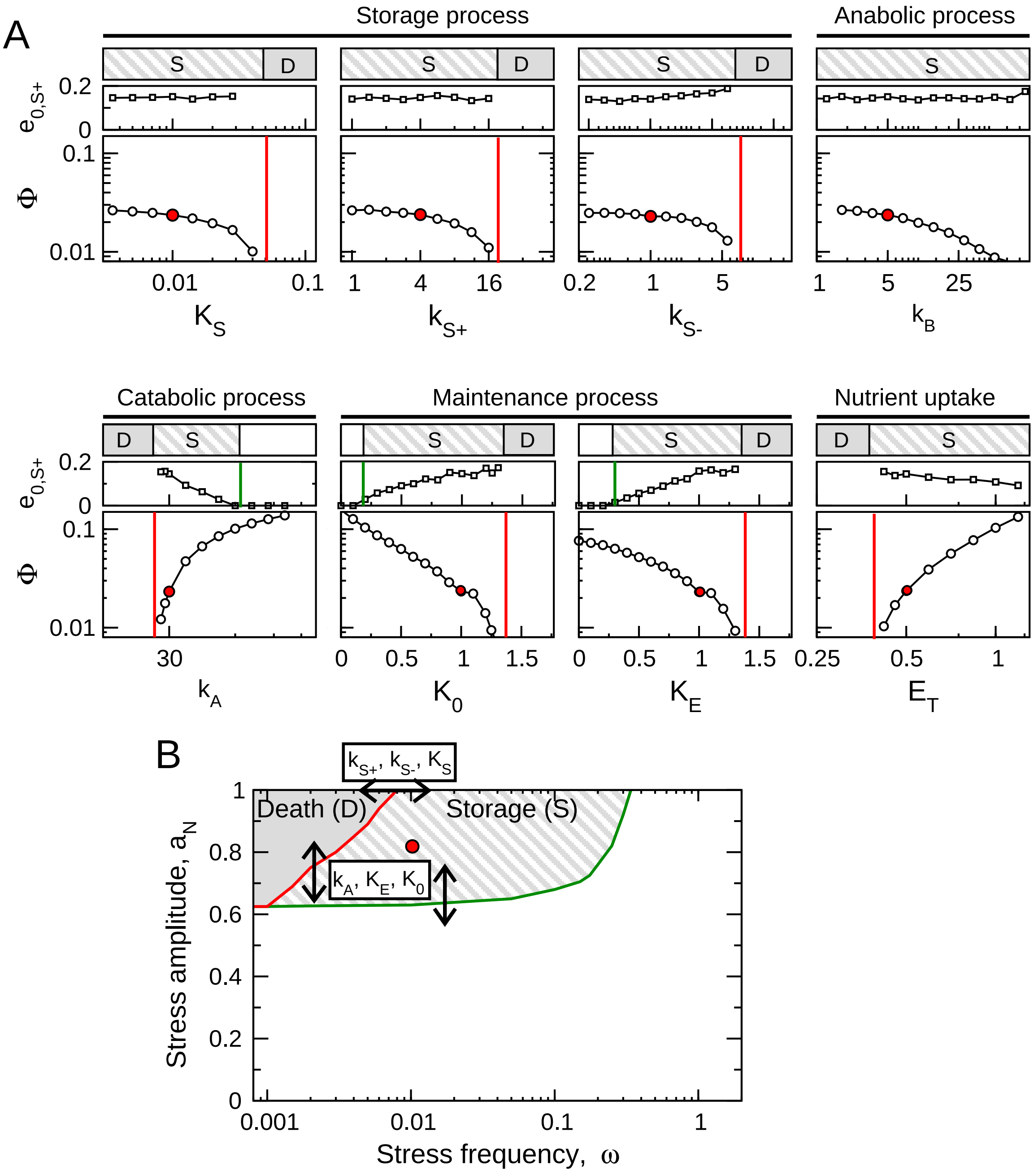

S3 Figure: Sensitivity analysis of non-optimized model parameters. (A) The analysis is performed for the optimal solution associated with the stress condition of amplitude $a_N = 0.8$ and frequency $\omega = 0.01$ (see Fig. 3A for the corresponding enzymatic parameter values). Growth rate score Φ and mean value of storage production enzyme $e_{0,S+}$ of optimized solutions as a function of the non-optimized model parameters k_{S+}, k_{S-}, K_S, k_A, K_E, K_0, which are varied independently in a log or linear scales. Upper horizontal bars indicate the corresponding metabolic regime (white: no storage, hatched grey: storage, S; grey: death, D). (B) Schematic representation of the influences of the non-optimized model parameters on the size and boundaries of the storage regime in the amplitude-frequency plane.
